# Supplementary material for: Production of Minor Ginsenosides from Panax notoginseng Flowers by Cladosporium xylophilum
Source: Molecules. 2022 Oct 5;27(19):6615. doi: 10.3390/molecules27196615 (PMC9572572; doi:10.3390/molecules27196615)
Supplement: Supplementary file 1 [file molecules-27-06615-s001.zip › molecules-1894187-supplementary.pdf]

Production of Minor Ginsenosides from *Panax notoginseng* Flowers by *Cladosporium xylophilum*

Table S1. ^{13}C NMR data for compounds **1**–**3** (notoginsenosides Fd, Fe and ginsenoside Rd_2) in C_5D_5N

Carbon No.	Fe	Rd_2	Fd	Carbon No.	Fe	Rd_2	Fd
	δ_C	δ_C	δ_C		δ_C	δ_C	δ_C
1	39.3	39.7	39.3		3-Glc	3-Glc	3-Glc
2	26.4	26.3	26.4	1′	106.7	105.7	106.7
3	88.5	88.6	88.5	2′	75.5	75.4	83.1
4	39.7	39.3	39.7	3′	78.4	79.0	78.1
5	56.0	56.0	56.0	4′	71.7	71.2	71.5
6	18.1	18.1	18.1	5′	78.1	78.0	78.1
7	34.7	34.8	34.7	6′	62.7	62.7	62.7
8	39.7	39.7	39.7		20-Glc	20-Glc	20-Glc
9	49.8	49.8	49.9	1″	97.8	98.0	97.8
10	36.6	36.5	36.6	2″	74.7	74.8	75.5
11	30.4	30.4	29.7	3″	78.4	78.0	78.4
12	69.9	69.9	70.4	4″	71.5	71.3	71.2
13	49.4	49.1	49.1	5″	76.3	76.9	76.9
14	51.1	51.1	51.0	6″	68.2	69.9	69.8
15	30.4	30.4	30.5		20-Ara(f)	20-Ara(p)	20-Xyl
16	26.3	26.4	26.4	1‴	109.9	104.8	105.7
17	51.3	51.3	51.1	2‴	83.2	71.5	74.6
18	16.3	17.4	15.9	3‴	79.0	77.6	78.3
18	15.6	16.3	16.3	4‴	85.5	68.4	71.1
20	83.2	83.0	83.0	5‴	62.3	65.4	66.7
21	22.0	22.1	22.6				
22	35.8	35.7	36.5				
23	22.8	22.9	23.2				
24	125.7	125.6	125.6				
25	130.7	130.6	130.7				
26	25.5	25.5	25.5				
27	17.6	15.9	18.1				
28	27.8	27.7	27.8				
29	16.5	17	16.5				
30	17.0	17.4	17.0				

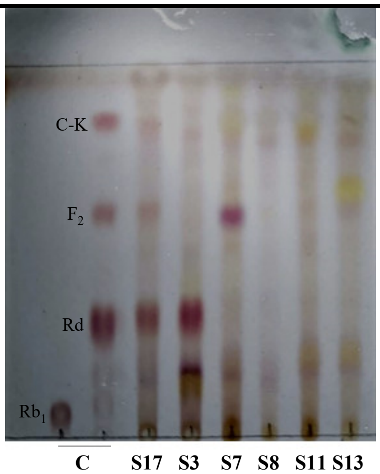

Figure S1. TLC analysis of the transformation of ginsenoside Rb_1 by different strains. C: authentic ginsensides (Rb_1, C-K, F_2, Rd); S17, S3, S7, S8, S11 and S13, transforming strains. (S17, S3 and S7 were *Pestalotiopsis biciliate*, *Coriolopsis gallica*, and *Cladosporium xylophilum*, respectively. Other 3 strains with lower transformation activity were not identified).

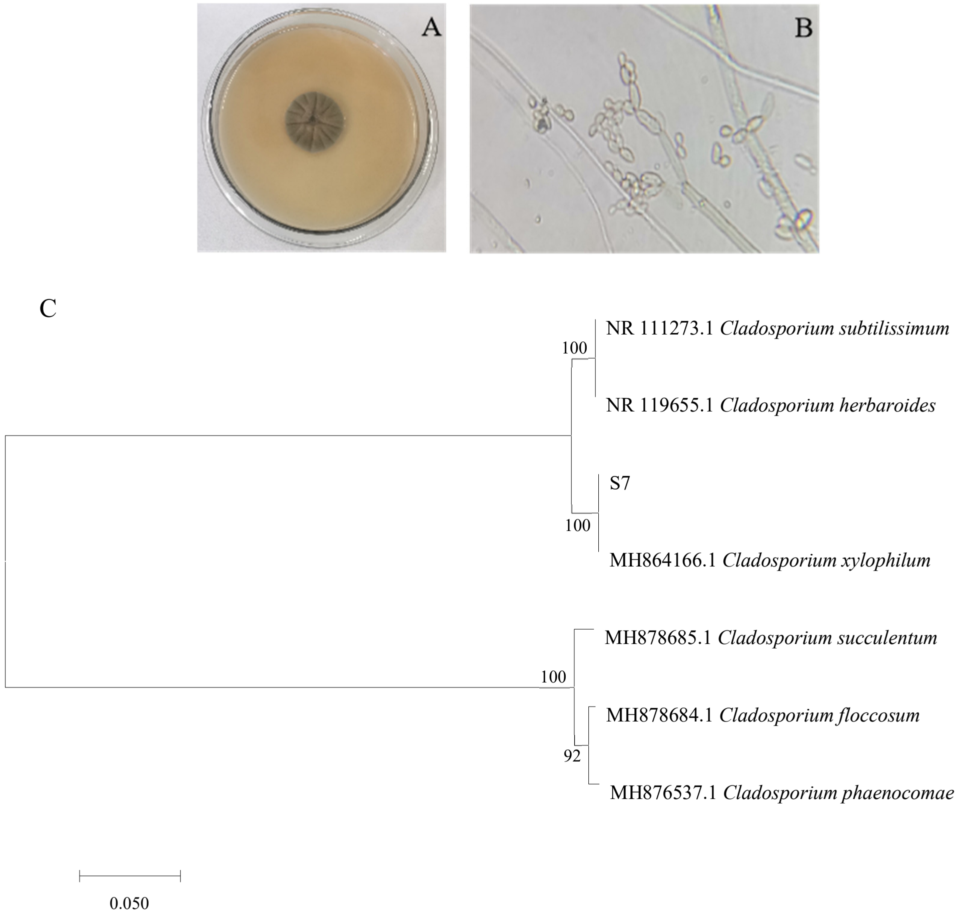

Figure S2. Morphology and ITS gene identification of strain-S7. (**A**) Colony morphology diagram; (**B**) Spore map of strain-S7; (**C**) The phylogenetic tree based on ITS rDNA gene sequences of strain-S7.

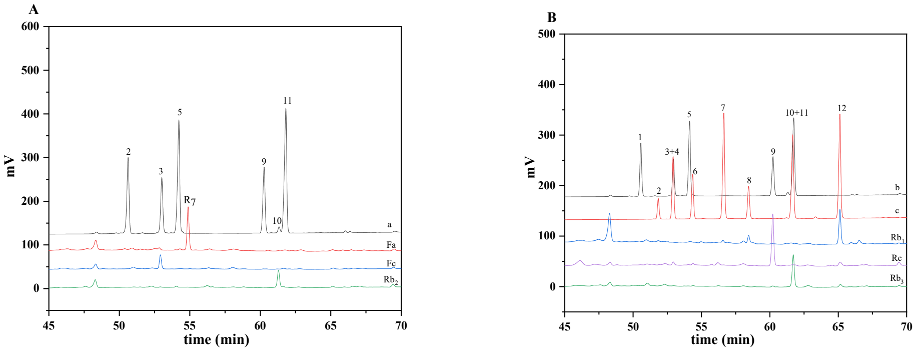

Figure S3. HPLC analysis of the transformation products of monomer ginsenosides Rb_1, Rb_2, Rb_3, Rc, notoginsenosides Fa and Fc by *C. xylophilum*. 12 authentic saponins (a,b,c). The peaks: notoginsenoside Fa (1); ginsenoside Rb_1 (2); notoginsenoside Fc (3); ginsenoside Rc (4); ginsenoside Rb_2 (5); ginsenoside Rb_3 (6); ginsenoside Rd (7); Gpy17 (8); notoginsenoside Fe (9); ginsenoside Rd_2 (10); notoginsenoside Fd (11); ginsenoside F_2 (12).

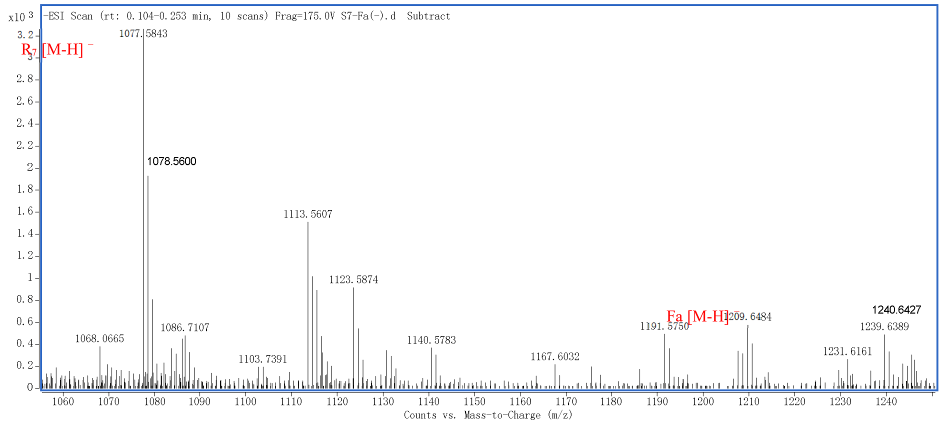

Figure S4. MS analysis of transformation products of notoginsenoside Fa by *C. xylophilum.*

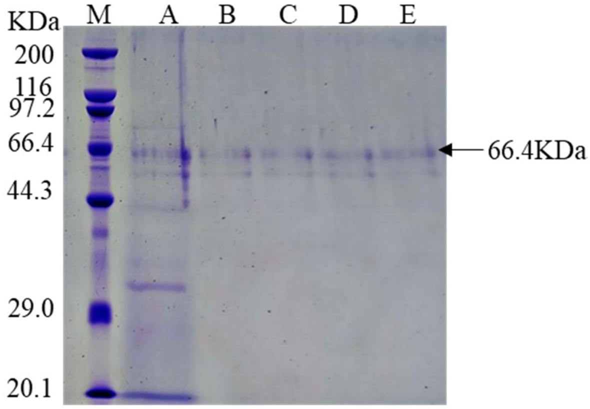

Figure S5. SDS-PAGE analysis of the purified *β*-glucosidase from *C. xylophilum* after protein staining with Coomassie Brilliant Blue solution. Protein marker (M). Crude extract (A). Purified enzyme (B–E).

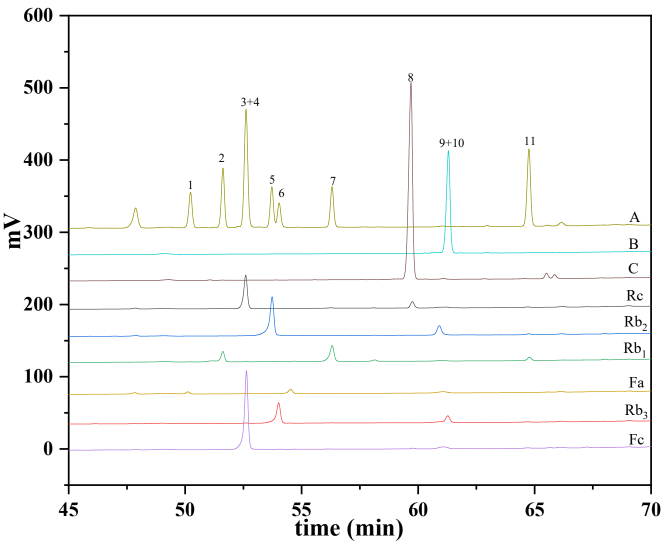

Figure S6. Biotransformation of ginsenosides Rb1, Rb2, Rb3, Rc, notoginsenosides Fa and Fc by crude enzymes. 12 authentic saponins (A, B, C). The peaks: Notoginsenoside Fa (1); ginsenoside Rb1 (2); notoginsenoside Fc (3); ginsenoside Rc (4); ginsenoside Rb2 (5); Ginsenoside Rb3 (6); Ginsenoside Rd (7); notoginsenoside Fe (8); ginsenoside Rd2 (9); notoginsenoside Fd (10); ginsenoside F2 (11). Rc, Rb2, Rb1, Fa, Rb3, and Fc were transformed substracts.

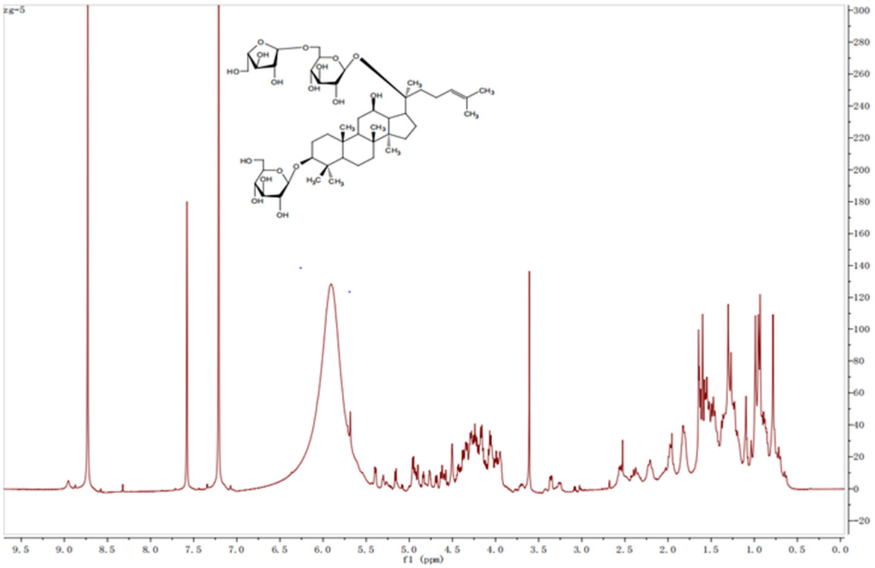

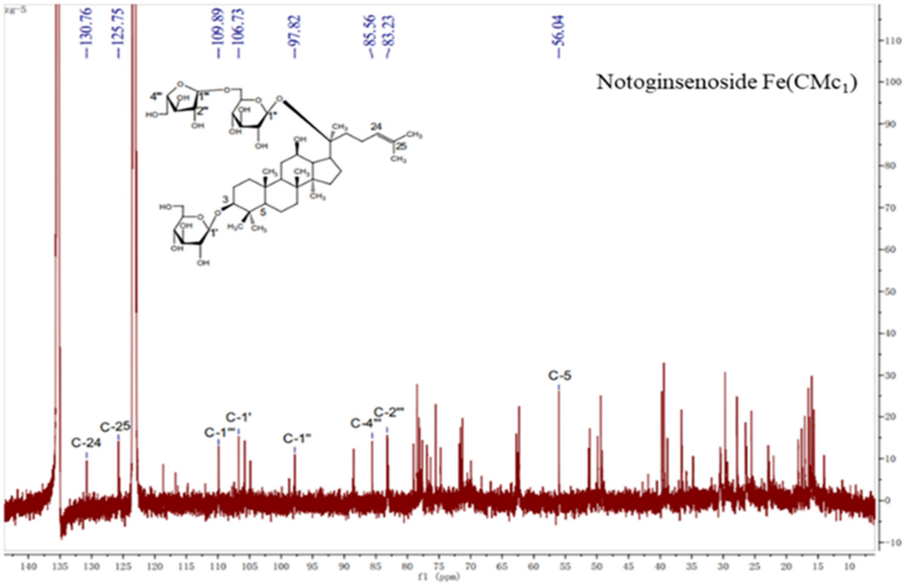

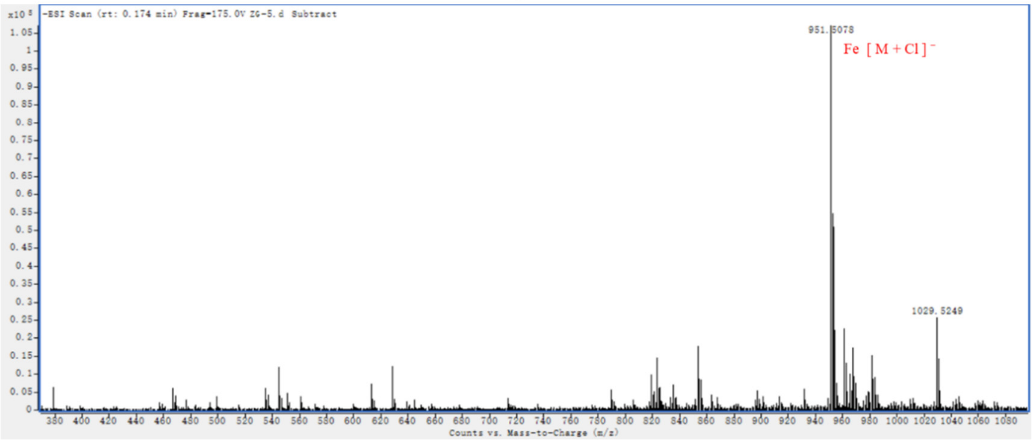

Figure S7. ^{1}H NMR, ^{13}C NMR (C_5D_5N) and MS spectra of compound **1.**

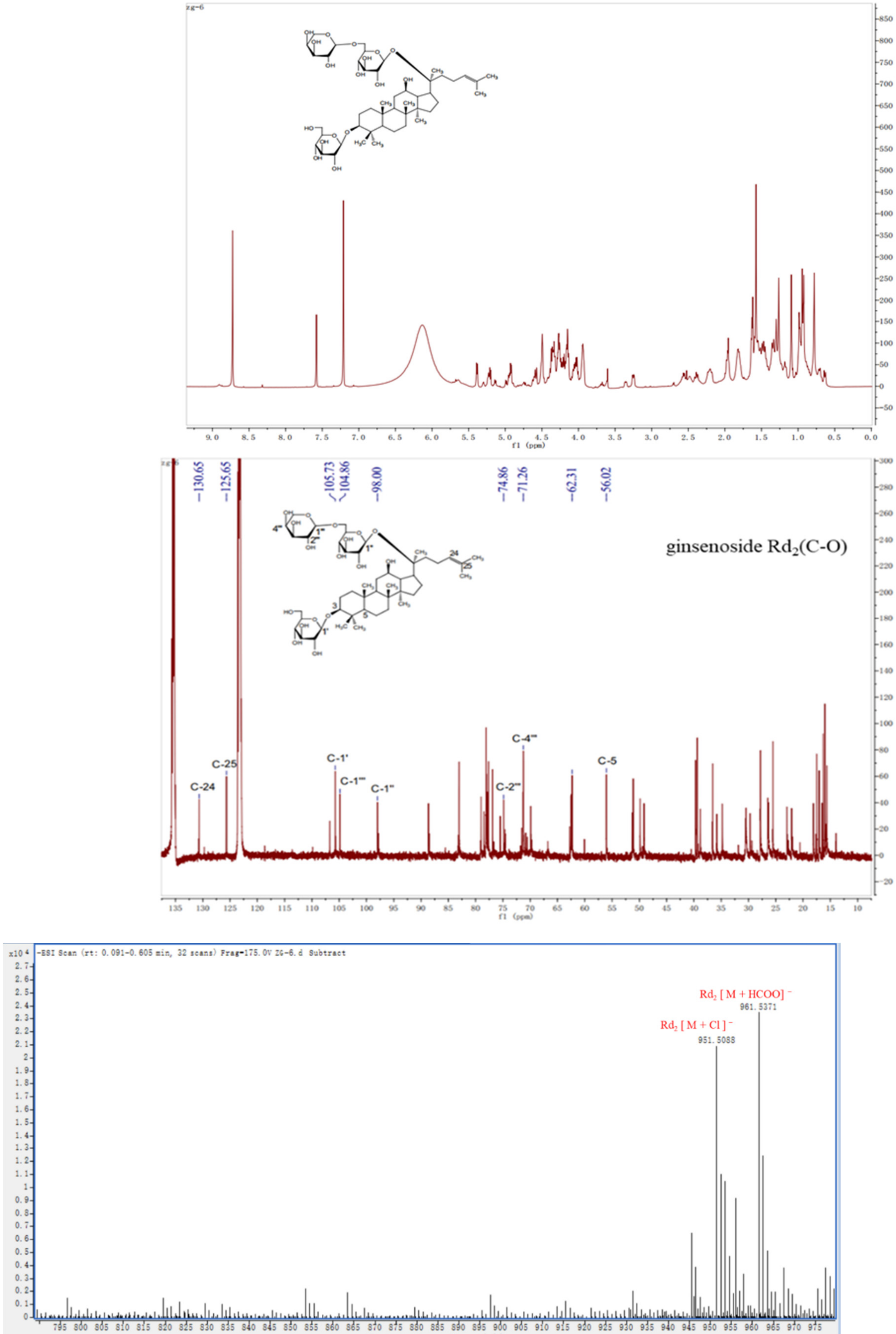

Figure S8. ^{1}H NMR, ^{13}C NMR (C_5D_5N) and MS spectra of compound **2.**

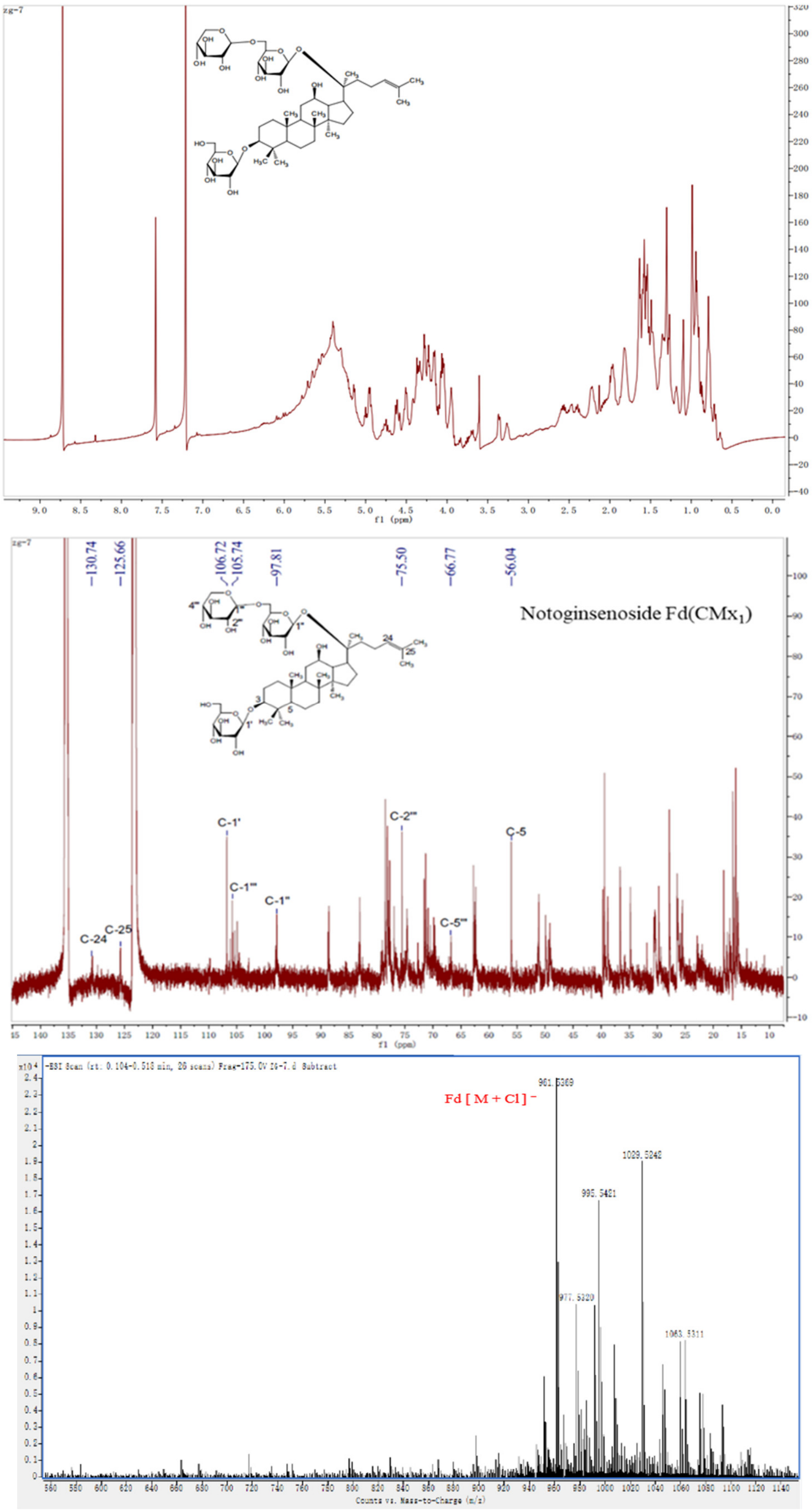

Figure S9. ^{1}H NMR, ^{13}C NMR (C_5D_5N) and MS spectra of compound **3**

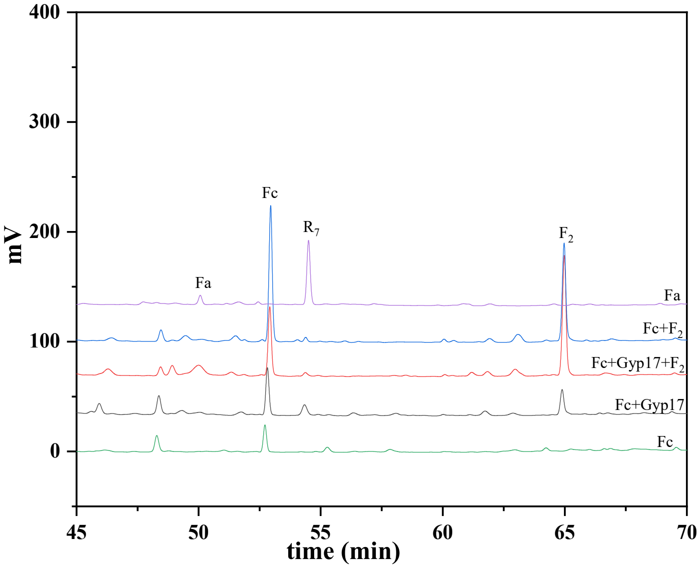

Figure S10. HPLC analysis of the transformation products of mixture of same mass of ginsenoside by *C. xylophilum*.
